# Metabolic Syndrome Is Associated with Increased Oxo-Nitrative Stress and Asthma-Like Changes in Lungs

**DOI:** 10.1371/journal.pone.0129850

**Published:** 2015-06-22

**Authors:** Vijay Pal Singh, Rangoli Aggarwal, Suchita Singh, Arpita Banik, Tanveer Ahmad, Bijay Ranjan Patnaik, Giridharan Nappanveettil, Kunal Pratap Singh, Madan Lal Aggarwal, Balaram Ghosh, Anurag Agrawal

**Affiliations:** 1 Centre of Excellence for Translational Research in Asthma and Lung Disease, CSIR- Institute of Genomics and Integrative Biology, Delhi, India; 2 National Centre for Laboratory Animal Sciences, National Institute of Nutrition, Tarnaka, Hyderabad, AP, India; 3 Shriram Institute of Industrial Research, University Road, Delhi, India; French National Centre for Scientific Research, FRANCE

## Abstract

Epidemiological studies have shown an increased obesity-related risk of asthma. In support, obese mice develop airway hyperresponsiveness (AHR). However, it remains unclear whether the increased risk is a consequence of obesity, adipogenic diet, or the metabolic syndrome (MetS). Altered L-arginine and nitric oxide (NO) metabolism is a common feature between asthma and metabolic syndrome that appears independent of body mass. Increased asthma risk resulting from such metabolic changes would have important consequences in global health. Since high-sugar diets can induce MetS, without necessarily causing obesity, studies of their effect on arginine/NO metabolism and airway function could clarify this aspect. We investigated whether normal-weight mice with MetS, due to high-fructose diet, had dysfunctional arginine/NO metabolism and features of asthma. Mice were fed chow-diet, high-fat-diet, or high-fructose-diet for 18 weeks. Only the high-fat-diet group developed obesity or adiposity. Hyperinsulinemia, hyperglycaemia, and hyperlipidaemia were common to both high-fat-diet and high-fructose-diet groups and the high-fructose-diet group additionally developed hypertension. At 18 weeks, airway hyperresponsiveness (AHR) could be seen in obese high-fat-diet mice as well as non-obese high-fructose-diet mice, when compared to standard chow-diet mice. No inflammatory cell infiltrate or goblet cell metaplasia was seen in either high-fat-diet or high-fructose-diet mice. Exhaled NO was reduced in both these groups. This reduction in exhaled NO correlated with reduced arginine bioavailability in lungs. In summary, mice with normal weight but metabolic obesity show reduced arginine bioavailability, reduced NO production, and asthma-like features. Reduced NO related bronchodilation and increased oxo-nitrosative stress may contribute to the pathogenesis.

## Introduction

Metabolic syndrome (MetS) and asthma are major global health concerns that have rapidly increased in preceding decades [1,2,3,4,5,6]. Asthma is characterized by reversible episodic airway obstruction with hyperresponsiveness, while MetS is a cluster of clinical symptoms such as obesity, insulin resistance, dyslipidaemia, hypertension, and glucose intolerance [7,8,9]. Studies indicate that metabolic syndrome is associated with lung function impairment that may manifest as new asthma or as decreased asthma control [10,11]. Among the component of metabolic syndrome that have been associated with asthma risk, obesity is the best understood [12,13]. While other components, such as hypertension and elevated glucose levels, have been shown to be important risk factors for the development of asthma, a mechanistic understanding of their link remains elusive [12,13,14,15,16]. Insulin resistance was found to be a stronger predictor for asthma like symptoms than increased body mass or waist circumference, in a Danish cohort [16], but recent analysis of the CARDIA study [17] suggested that the risk for new asthma is mostly attributable to increased body mass in women. It is therefore unclear whether metabolic changes of obesity, referred to as metabolic obesity, are independent risk factors for asthma. This is important because in many parts of the developing world, individuals with normal body mass index (BMI) commonly exhibit metabolic obesity. If such individuals are at increased risk of asthma, asthma incidence will rise sharply in years to come. Yet, experimental understanding of the influence of MetS on lung function, independent of obesity, is lacking. Studies of lung function and asthma features in obese and non-obese models of MetS could be helpful.

While some obese patients with allergic asthma have more severe inflammatory disease than their lean counterparts [18,19,20], there also appears to be a distinct “obese-asthma” phenotype [21] where severity is independent of cellular inflammation [22,23,24,25,26,6,27]. Thus, it seems possible that pathways unrelated to classical immune response may be altered that are specific to the obese/metabolic syndrome state giving rise to an obese-asthma phenotype different from conventional Th2-mediated inflammatory pathways. In both mice and humans, allergic airway inflammation (AAI) is found to be positively correlated with enhanced exhaled nitric oxide (ENO) [28,29]. However, the human obese- asthma phenotype is characterized by low exhaled nitric oxide, absence of typical allergic inflammation, poor response to steroids, and poor symptom control [30]. In human studies, plasma ratio of L-arginine/asymmetric dimethylarginine (ADMA) has been found to explain the inverse relationship between BMI and exhaled NO in late-onset asthma phenotype [31].

It is known that asymmetric dimethylarginine (ADMA) competes for binding to Endothelial Nitric Oxide Synthase (eNOS) with L-arginine [32]. Binding of eNOS to ADMA leads to its uncoupling such that reactive oxygen species are generated. Inducible NOS (iNOS), induced by inflammatory stimuli, synthesizes high levels of NO, which, together with reactive oxygen species, leads to the generation of reactive nitrogen species [33]. Moreover, alteration in NO has also been related to mitochondrial dysfunction in asthmatic lungs [33,34].

Here, to understand the consequence of normal-BMI metabolic obesity upon the lungs, we used high-fat (obese) and high-fructose (non-obese) diet induced mice models of MetS. This allows us to experimentally segregate the effects of obesity from other components of MetS and clarify the mixed epidemiological human data on the subject [10, 12, 13, 14, 15, 16]. We placed particular emphasis on dissecting the arginine/NO metabolism in these studies because it offers an attractive mechanism for a link between MetS and asthma. While high-fat diet induced mouse models of obesity have previously been investigated for the presence of AHR [35], there is no such data on non-obese models of metabolic syndrome. We show for the first time that diet-induced dysfunctional arginine metabolism is associated with asthma-like changes, independent of obesity, suggesting that metabolic obesity in BMI-normal individuals is an important risk for asthma.

## Materials and Methods

### 2.1 Animals and diet plan

Four to five week old male C57BL/6 mice were obtained from National Institute of Nutrition (Hyderabad, India) and acclimatized for a week prior to starting the experiments. All animals were maintained as per CPCSEA (Committee for the Purpose of Control and Supervision of Experiments on Animals) guidelines and protocols were approved by Institutional Animal Ethics Committee.

After one week of acclimatization mice were divided in to three groups, six in each (n = 6) and were named according to diet provided as Control, HFA and HFR and were housed in IVC (Individually Ventilated Cages; 25±2°C, 50% humidity; 12:12hr dark/light cycle) with enrichment facilities. The mice had free access to either a standard rodent chow (CN) (5.5% fat and nil refined fructose), or a high-fat diet (HFA) having 60% of energy from fat, or a high fructose diet (HFR) with 70% energy from fructose. The high fat and high fructose diet were obtained from Research diet Inc. U.S.A.

*Ad libitum* amount of feed and purified water was provided for 18 weeks. Fresh diet was provided daily and regular intake of diet of mice was observed to keep in check the proper intake of food. Daily visit of at least half an hour were made to animals to acclimatize mice with handler and normal behavioural elements such as grooming, walking, exploring, scratching, stretching and proper eating and drinking were observed on regular basis. Every week, weight estimation was done to monitor the weight gain. At eight weeks age, mice were given unique codes for individual identification with the help of Radio frequency identification device (RFID). During the 17th week of diet Body mass assessment, Blood pressure measurement and Exhaled nitric oxide measurement were done in all mice of different diet fed groups.

The method for the sacrifice of animals used was the combination of xylazine and thiopentone sodium as per body weight. In combination, xylazine acts as an analgesic and thiopentone sodium as anaesthesia which relaxes muscles and anesthetizes mice. This combination minimizes the suffering and also it is as per ethical guidelines used for euthanasia of laboratory animals.

### 2.2 Fat mass and lean body mass assessment by dual energy X-ray absorptiometry

We assessed body fat mass and lean mass by DXA, (Hologic, QDR model). Mice scanned alive were anesthetized with 3% isoflurane which were then placed onto the exposure platform of the machine. Automatic scan of animal and analysis of data was done. The raw scan data containing the attenuation values of tissue were captured and transferred to computer. Algorithm software interprets each pixel and creates quantitative measurement of the body tissues. The data was finally retrieved from the computer.

### 2.3 Non invasive blood pressure measurement

Blood pressure was measured with Non Invasive Blood Pressure measurement technique (IITC, NIBP Multi channel Blood pressure system), a computerized tail-cuff system for measuring blood pressure in small animals such as mice. After restraining the animal, a cuff was placed around the tail of the animal such that the sensor faced caudal artery and was then inflated. This caused the pulsations at a more distal and pulse sensor ceased, as the cuff was slowly deflated the reappearance of pulsation was noted and the cuff pressure at which this occurred was taken to be systolic in the tail. The results were displayed as data plots and were also permanently recorded in the computer files.

### 2.4 Measurement of exhaled nitric oxide

Exhaled NO was measured indirectly from a mouse placed inside a whole body plethysmograph (Buxco, USA) using a standard clinical photometric ENO analyzer (CLD88sp,Ecomedics, measurement range of 0.01–1,000 ppb), as described previously [28].

### 2.5 Airway hyperresponsiveness measurement

After 18 weeks of diet, mice were anesthetized first with Xylazine (16mg/kg body weight) for 5 minutes and then with thiopentone sodium (50mg/kg body weight) for another 2–3 minutes. After the mice were fully anesthetized, trachea was opened and mice were cannulated and put on the ventilator (Scireq, Canada). Positive end expiratory pressure (PEEP) was fixed at 2 cm H_2_O. On the ventilator TLC manoeuvres were first run in order to open any obstruction in the airways, before starting the measurements. Lung resistance and elastance were measured.

### 2.6 Collection of blood and measurement of blood glucose

Blood samples were rapidly obtained by cardiac puncture. A blood glucose meter (Accu-chek Active) was used for random quantitative determination of blood glucose values from fresh blood approximately 2 μl, using Accu-chek Active test strips. Serum was then isolated from remaining blood and stored at -20°c.

### 2.7 Biochemical analysis

Triglyceride and Cholesterol estimation was done in serum samples by Quantitation Kits (BIOVISION) as per the manufacturer’s instructions. Serum insulin levels were measured by a commercially available ELISA kit (Millipore) as per the instructions. HOMA-IR was calculated as [blood glucose (mg/dl) × insulin μU/ml)]/405 [36].

### 2.8 Measurement of asymmetric dimethylarginine (ADMA) by a novel ELISA method

Total lung protein fractions and serum samples taken in duplicates were used for ADMA ELISA (Diagnostika, GMBH, Hamburg, Germany). ADMA levels were measured by a novel ELISA method, which uses the microtiter plate format. ADMA is bound to the solid phase of the microtiter plate, ADMA in the samples is acylated and competes with the solid phase bound ADMA for a fixed number of rabbit anti-ADMA antiserum binding sites. The antibody bound to the solid phase ADMA is detected by anti-rabbit/peroxidase. The substrate TMB/ peroxidase were used. Results were expressed in nanomoles and normalized by protein concentrations.

### 2.9 Western blot analysis

For Western blot of eNOS and iNOS, total lung proteins were separated by 10% SDS-PAGE, transferred onto PVDF membrane. Transferred membrane was blocked with blocking buffer (3% Bovine Serum Albumin in PBS with Tween 20), incubated with eNOS and iNOS antibody (1:500;abcam), followed by horseradish peroxidase–conjugated secondary antibodies (anti-rabbit for eNOS and iNOS; Sigma), and detected with DAB-H_2_O_2_ (Sigma). α-Tubulin was used as a loading control. Similarly, western blot of arginase I was performed in lung cytosolic fraction protein with arginase I antibody (1:500, Santa Cruz biotechnology). β actin was used as a loading control. Signals were detected by spot densitometry (Alpha EaseFC software from Alpha Innotech).

### 2.10 Immunohistochemistry

Commercial goat polyclonal antibodies for eNOS, iNOS and arginase I were used as primary antibodies and respective HRP conjugated secondary antibodies (Sigma) were used for Immunohistochemistry and were performed as described previously [37]. IHC profiling using IHC profiler plugin in ImageJ software was done for iNOS and arginase I for more distinguishable and differential analysis of DAB and Haematoxylin uptake by different group tissues. [38]

### 2.11 Estimation of nitrative stress in lungs

The levels of nitrotyrosine, a marker of nitro-oxidative stress, were measured in lung homogenates by competitive ELISA method (Cayman, An Arbor, Michigan, USA), and results were expressed in nanomoles/10μg protein.

### 2.12 Lung histopathology

Formalin fixed, paraffin embedded, lung tissue sections were stained with Haematoxylin & Eosin (H & E) and Masson’s Trichrome (MT) staining to assess the airway inflammation, and sub-epithelial collagen content respectively as described previously [39]. Stained sections were observed and microphotographs were taken with Nikon microscope with camera (Model YS-100). Collagen deposition in MT-stained skin sections was estimated by quantitative morphometry as described previously [39].

### 2.13 Measurement of arginine level in lungs by reversed phase high performance liquid chromatography method

To determine the level of arginine in mice lungs, three mice from each group were taken and their lungs were surgically excised, weighed and pooled. Lungs were immediately homogenized in liquid nitrogen followed by addition of ice cold cell lysis buffer. Homogenized tissue was incubated for 1 h on ice and centrifuged for 15 min at 4°Celsius at 15,000× g. The resulting supernatant was collected and proteins were precipitated by adding an equal volume of 20% (v/v) trichloroacetic acid. The samples were again centrifuged at 15,000× g for 12 min, the supernatants were removed and pellets collected. The protein pellets were washed with 100 μL ice-cold acetone for 60 min at -20°C. The suspension was centrifuged at 15,000× g for 12 min at 4°C and the resulting protein pellet was dissolved in 100 μL HPLC grade water. Prior to protein hydrolysis, total protein estimation by BCA method was performed. Gas-phase hydrolysis with 6M HCL, 110°C for 16h was used for total hydrolysis of precipitated protein fractions. Vacuum centrifuge was used to dry the samples. Samples were stored at -20°C. For sample and standard (L-arginine) derivatization: 0.05 ml of sample/standard was mixed with 0.450ml of derivatization reagent (7:1:1:1; mixture of ethanol, water, triethylamine and phenyl-isothocynate; freshly prepared). This was incubated at 25°C for 25 minutes. The final volume was adjusted to 1ml with diluent (Disodium hydrogen sulphate buffer pH-7.4). This was filtered through 0.2μ syringe filter and injected in to the HPLC system. The derivatives were separated on a C18 Phenomenex column (250×150 mm; 5 μ pore size) at 30°C and a flow rate of 1.0ml/min. The derivatives were detected at 254nm. Samples were run in duplicates.

### 2.14 Statistical analysis

Data are expressed as means (±SD). Differences between the groups were analyzed with the Student t test for two groups, or ANOVA for more than two groups, and a p value ≤0.05 was considered as statistically significant.

## Results

### 1. The obese and non-obese model of metabolic syndrome in mice

**Fig 1:**
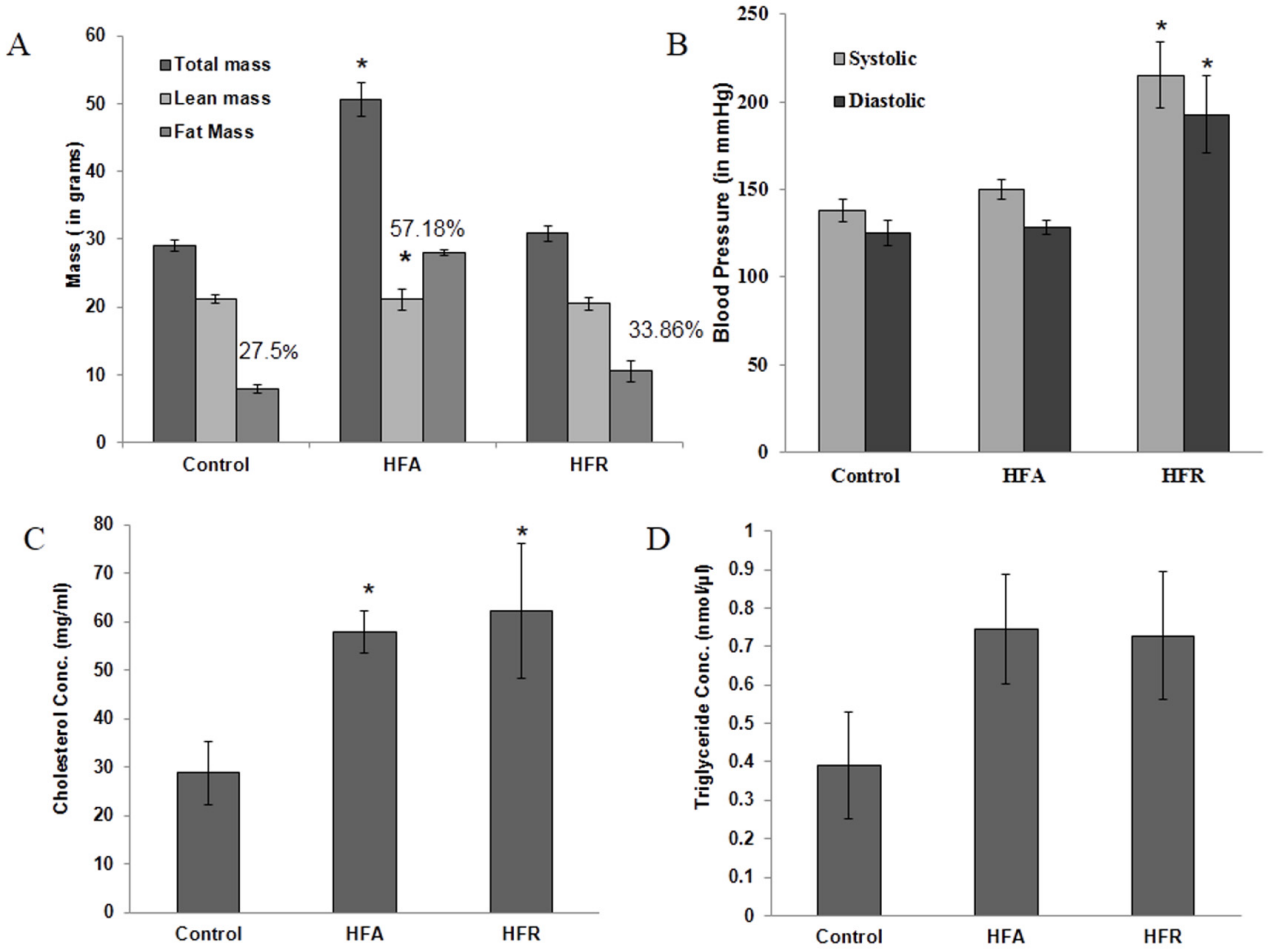
The obese and non-obese model of metabolic syndrome in mice. (A) Total mass, Lean mass, Fat mass and % Fat in control, high fat and high fructose diet groups. (B) Cholesterol levels in blood serum from Control, HFA and HFR diet mice groups. (C) Triglycerides levels in blood serum from Control, HFA and HFR diet mice groups. Data shown here are Mean ± SE of 6 mice in each group.*Denotes statistically significant differences (p<0.05) vs. Control.

To determine the effects of high fat or high fructose diet on components of metabolic syndrome, we first measured the change in total mass and fat mass in different dietary groups as mentioned in methods. While HFA diet led to more than 40% increase in total body mass (p<0.05), the HFR diet was not associated with weight gain, when compared to mice fed a normal chow diet (CN). While lean mass was not significantly different between groups, fat mass was more than doubled in the HFA group. Percent body fat was significantly higher in the HFA mice compared to either HFR or control mice on normal diet (Fig 1A). To determine the effect of different diets in inducing a state of dyslipidaemia; serum cholesterol and triglyceride levels were also measured. Both HFA and HFR diets were found to be associated with a significant increase in serum cholesterol (Fig 1C) and a trend towards increased serum triglycerides (Fig 1D), indicating dyslipidaemia. Increase in serum insulin levels as well as random blood glucose levels, with increased HOMA –IR values (Table 1) indicated insulin resistance and dysglycemia in HFA and HFR groups as compared to CN group. Non-invasive blood pressure from tail cuff was found to be significantly elevated in HFR group (Fig 1B).

**Table 1:** Mean± SEM is shown.

Groups	Control	HFA	HFR	
N	6	6	6	p Value
**Glucose (mg/dl)**	201±58	383.33±26	451.33±46	<0.03
**Insulin (ng/ml)**	0.32±0.04	0.67±0.10	0.44±0.03	<0.02
**HOMA-IR**	3.03 ±0.7	14.57±3.0	10.71±0.9	<0.01

HOMA-IR was calculated as [glucose (mg/dl) × insulin μU/ml)] /405.

* indicates statistically significant differences vs. Control (p<0.05).

### 2. Increased baseline resistance and decreased exhaled NO in mice with metabolic syndrome

**Fig 2:**
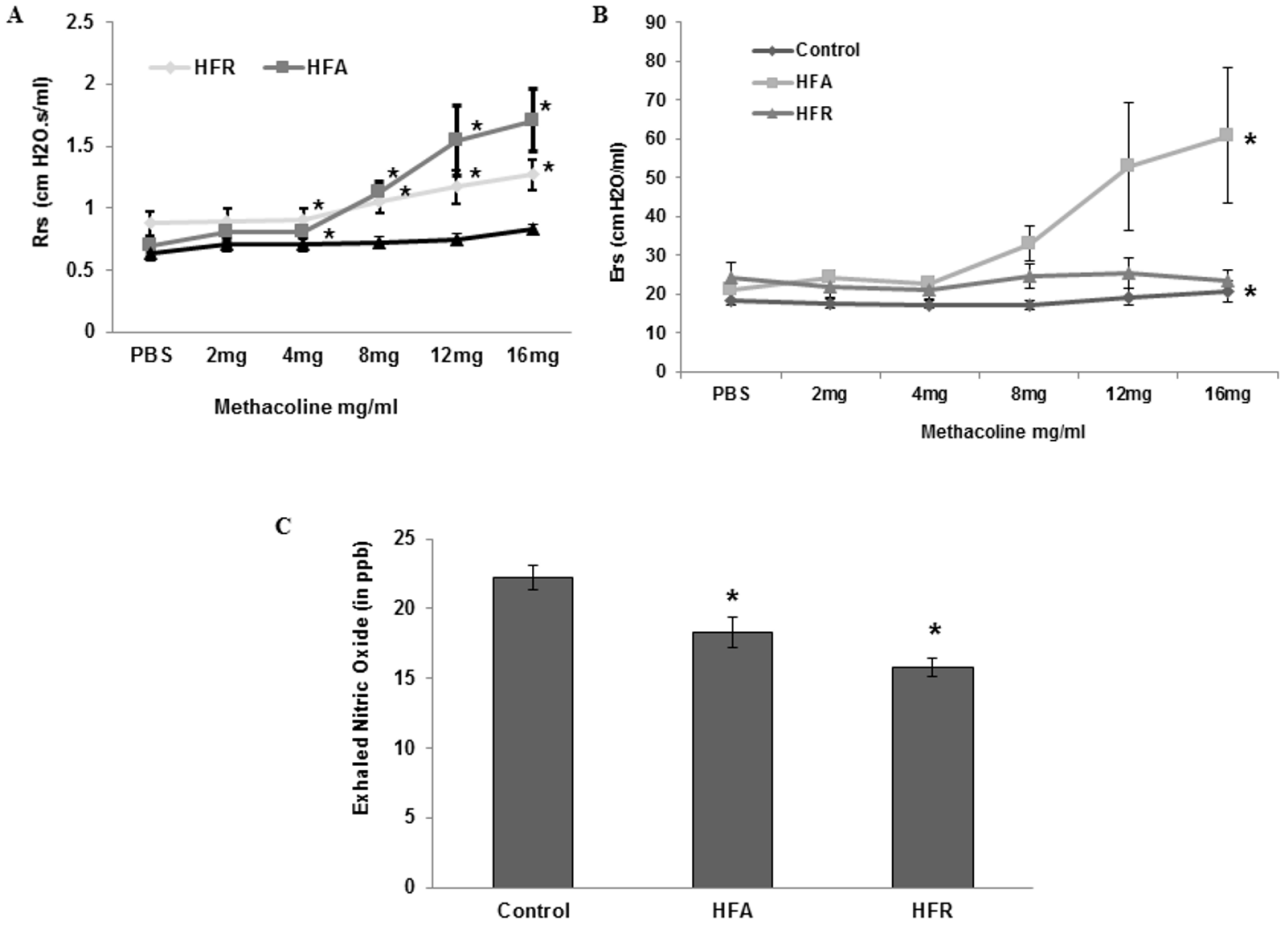
Increased baseline resistance and decreased exhaled NO in mice with metabolic syndrome. (A-B) Baseline lung resistance (R) and elastance (E) in anesthetized and ventilated control, high fat (HFA) and high fructose (HFR) diet fed mice (Mean ± SE, n = 12 per group, two independent sets of experiments). (C) Non-invasively measured exhaled NO of control, HFA and HFR mice (n = 6 per group). All data are Mean ± SE. n = 6 mice in each group *Denotes statistically significant differences (p<0.05) vs control.

Baseline lung resistance was found to be increased in HFR group, compared to CN mice. Maximal lung resistance after methacholine challenge was increased in both HFR and HFA mice and was maximal in HFA group (Fig 2A). Elastance was similar at baseline but went up significantly in HFA mice during methacholine challenge (Fig 2B), presumably due to greater peripheral airway closure in obese mice at identical PEEP settings. Since reduced exhaled NO has been previously observed in obese human asthmatics [22], we further measured exhaled NO (ENO) in a subset of each group. Significantly lower levels of ENO were found in HFA and HFR groups compared to CN (Fig 2C).

### 3. High-fat or high-sugar diets lead to reduced arginine bioavailability and increased ADMA associated oxo-nitrative stress

**Fig 3:**
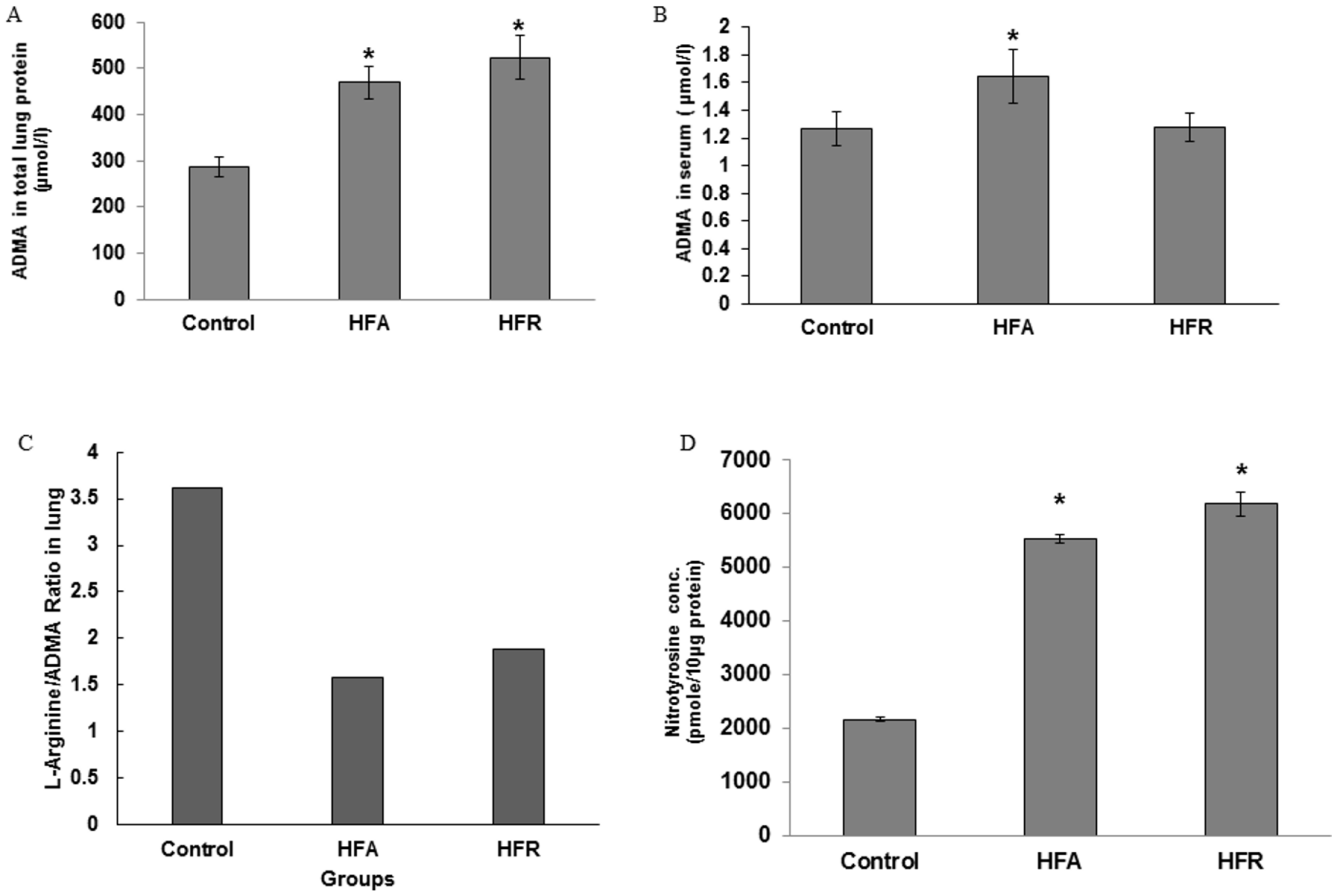
High-fat or high-sugar diets lead to increased ADMA associated oxo-nitrative stress and organelle dysfunction, independent of obesity. (A) ADMA levels were estimated from CN, HFA and HFR mice in total lung protein (TLP). (B) L-Arigine/ADMA ratio in lung. (C) ADMA levels in serum. (D) Nitrotyrosine levels in lung cytosolic fraction. All data are Mean ± SE. n = 6 mice in each group.*Denotes statistically significant differences (P<0.05) vs. Control.

To determine whether reduced exhaled NO was related to reduced L-arginine availability and uncoupling of Nitric-Oxide-Synthase (NOS) by asymmetric dimethyl arginine (ADMA), we measured L-arginine levels in mice lungs and ADMA levels in serum and lungs of mice from all groups. The L-arginine level in pooled lungs of HFA mice (150.44 ppm by HPLC) was lower than that of CN mice (209.38 ppm) or HFR mice (199.55 ppm). ADMA was significantly increased in the lungs of HFA and HFR mice, compared to CN mice (Fig 3A). ADMA levels were also increased in serum of HFA mice compared to CN mice (Fig 3B). The lung L-arginine/ADMA ratio, reflecting local arginine bioavailability for NO production, was therefore reduced in both models of metabolic syndrome (Fig 3C). Other than reducing availability of L-arginine, ADMA has also been postulated to play a major role in asthma, where it significantly contributes to oxo-nitrative stress via generation of superoxide anions (O2−) by uncoupled NOS, which reacts with NO in the airway cells leading to the formation of peroxynitrite, a highly reactive oxidant species [40]. We thus hypothesized that increased levels of ADMA may be responsible for oxo-nitrative stress and organelle dysfunction. Since peroxynitrite is unstable, but reacts with tyrosine residues in proteins to form the stable product nitrotyrosine, we measured nitrotyrosine levels in lung homogenates by competitive ELISA method. Nitrotyrosine was increased in HFA and HFR mice, compared to CN mice (Fig 3D).

**Fig 4:**
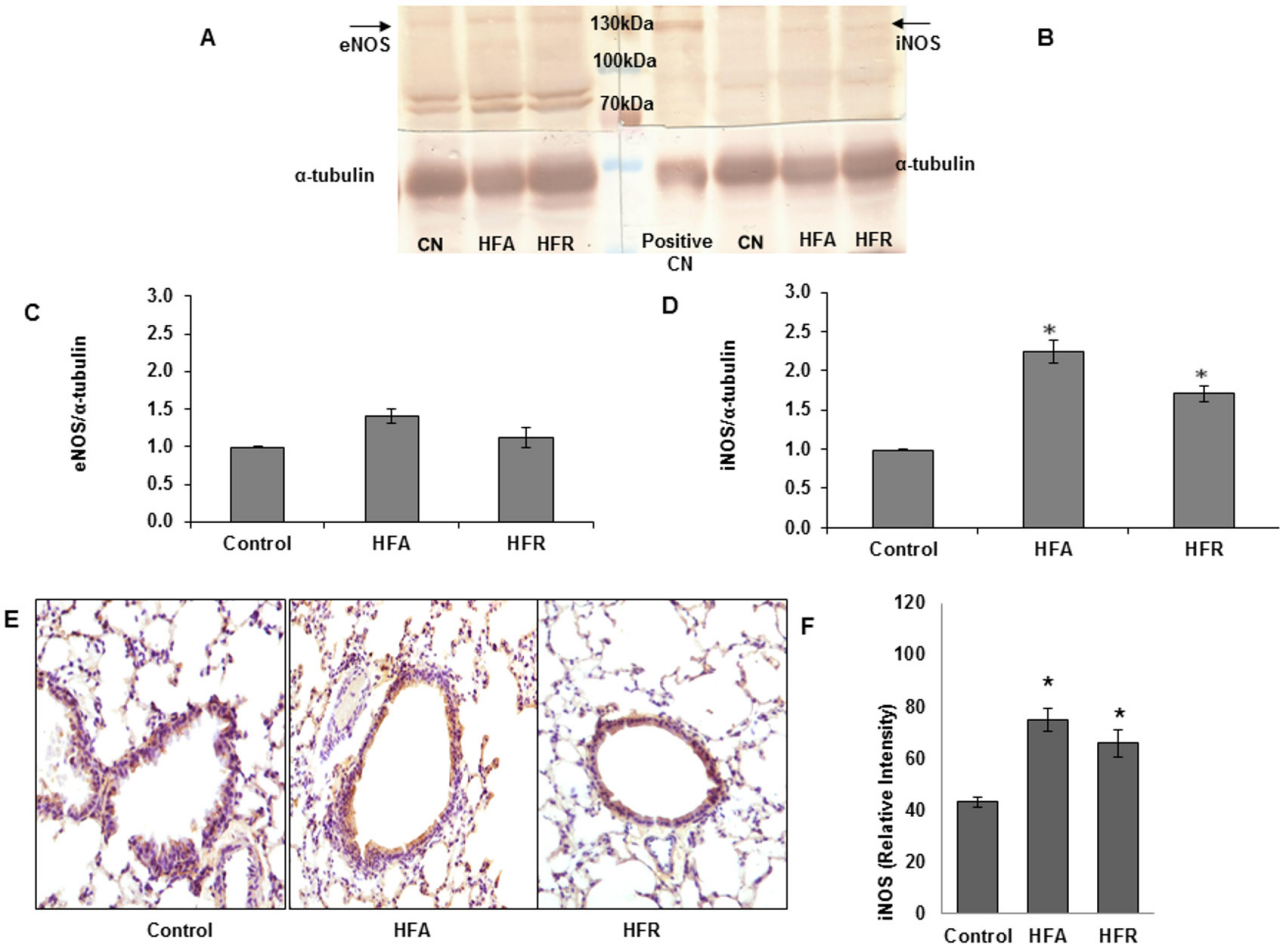
Induction of Inducible Nitric oxide synthase (iNOS) in lungs of mice with metabolic syndrome. (A) Western blot analysis of eNOS. (B) Western blot analysis of iNOS (C) Densitometry of eNOS (D) Densitometry of iNOS (E) Immunohistochemistry of iNOS. Brown colour indicates the positive expressions. HFA and HFR diet fed mice showed high expression of iNOS as compared to Control mice. Representative images are shown from each group. All photographs are at 10X magnification. Scale bar = 100μm. (F) Quantitative analysis for iNOS done using ImageJ software showed significant increase in its expression in HFA and HFR mice as compared to CN.*Denotes statistically significant differences (P<0.05) vs. Control.

**Fig 5:**
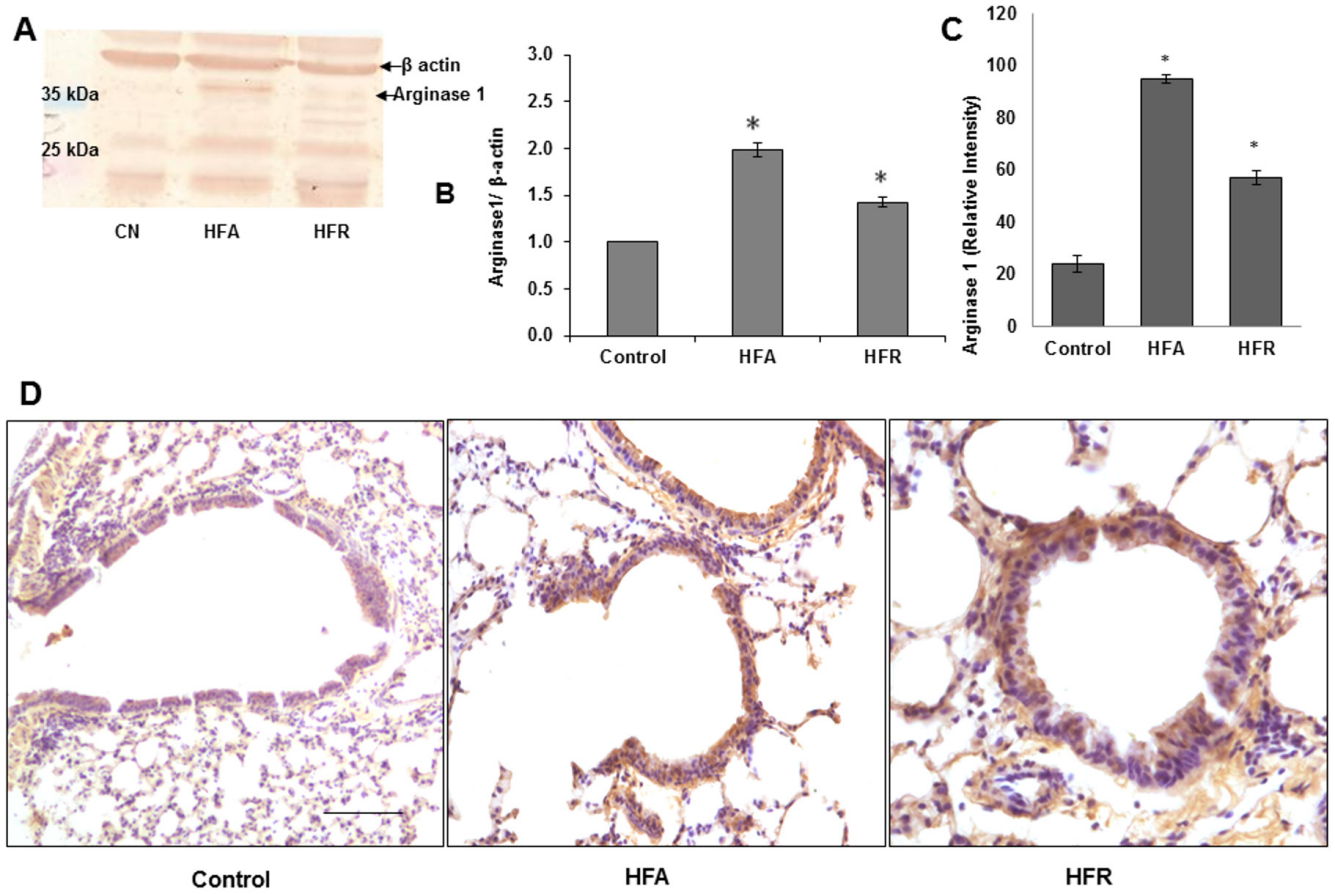
Increase in arginase levels in lungs of mice with metabolic syndrome. (A) Western blot analysis of arginase 1 (B) Densitometry of arginase 1 (C) Quantitative morphometry for arginase 1 IHC (D) Immunohistochemistry of arginase, Brown colour indicates positive expression. Representative images are shown from each group. All photographs are at 10X magnification. Scale bar = 100μm. *Denotes statistically significant differences (P<0.05) vs. Control.

We further investigated whether enzymes that regulate arginine metabolism and NO production (arginase-1, eNOS, and iNOS) were differently expressed in lungs of mice with MetS. No major change was seen in eNOS expression (Fig 4A and 4C) and iNOS expression was somewhat increased (Fig 4B–4F, S2 Fig), suggesting that the reduction in NO production was related to functional inhibition of the NOS pathway and not loss of NOS enzymes. In HFA group, lung arginase-1 expression was markedly increased consistent with lower L-arginine levels in lungs of such mice (Fig 5A–5C) (see above). Minimal increase in Arginase-1 was also seen in HFR (Fig 5D, S1 Fig).

### 4. Lack of cellular inflammation in lungs of mice with metabolic syndrome

**Fig 6:**
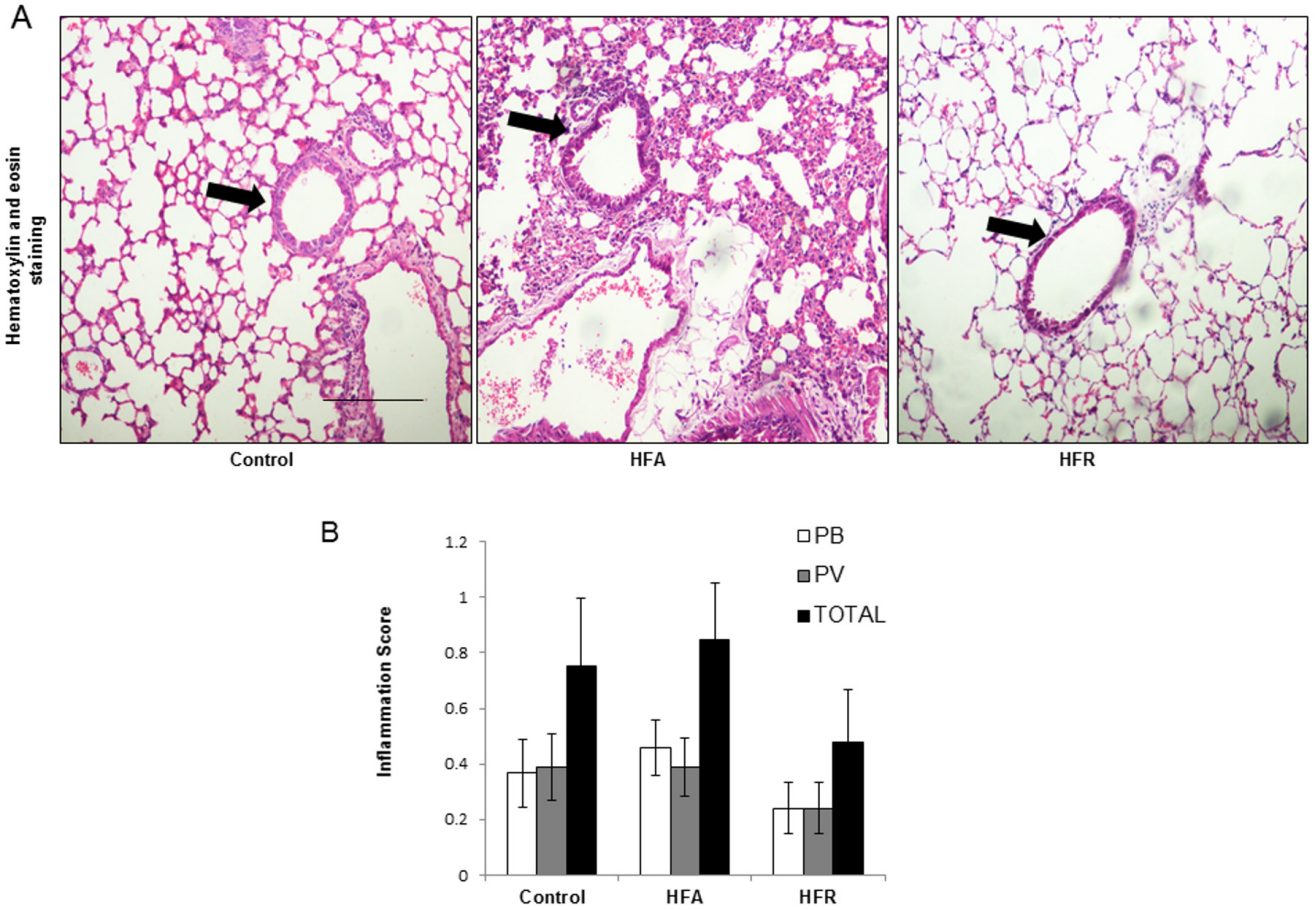
Lack of cellular inflammation in lungs of mice with metabolic syndrome. (A) Lung sections were stained with haematoxylin and eosin to estimate airway inflammation. (B) Inflammation score of the lungs was evaluated by experimentally blind experts and shown as perivascular (PV), peribronchial (PB) and Total (sum of both PV and PB). (Representative images are shown from each group. All photographs are at 10X magnification. Scale bar = 100μm.

To determine the effects of increased oxo-nitrative stress and other metabolic changes on lung structure, we performed histological studies. Cellular infiltration was measured with Haematoxylin & Eosin staining. Inflammatory cells did not appear to be significantly different between groups (Fig 6A) and this was also confirmed by a semi-quantitative inflammation score of the lungs (Fig 6B). Sub epithelial collagen, as judged by Masson Trichrome stain, appeared to be increased in HFA and HFR mice (S3A Fig). However, quantitative morphometry of the sub-epithelial collagen content showed high variability and the differences between groups were not significant by ANOVA (S3B Fig). However, the pooled MetS groups (HFA and HFR) had significantly greater sub-epithelial collagen content than control mice (one-tailed t-test, p = 0.0256).

## Discussion

The association of obesity with asthma is now well known, with many mouse and human studies confirming this link. There is some evidence that obesity is just one of the many components of the metabolic syndrome that may better explain asthma risk [12]. Studies across geographical regions and age-groups support a link with either the overall metabolic syndrome or its components such as hypertriglyceridemia and hypertension [41,15,42]. However, in an analysis of the CARDIA study by Assad et al, risk of incident asthma in women with metabolic syndrome became statistically insignificant after adjustment for BMI [17]. It therefore remains unclear whether normal-weight subjects with metabolic obesity are at increased risk of asthma. Such studies are lacking even in mice.

We chose to address this gap in knowledge by manipulating diets of C57BL/6 mice to induce metabolic syndrome, independent of obesity or adiposity. A high fat diet in these mice was associated with obesity, adiposity, hyperlipidaemia and hyperglycaemia; while a high fructose diet led to hyperlipidaemia, hyperglycaemia, and hypertension, but no obesity or adiposity (see Fig 1 and Table 1). These extreme diets mimic “junk foods” and induce a metabolic syndrome like state with or without obesity, permitting us to determine experimentally whether obesity is essential for development of asthma-like changes in lungs.

**Fig 7:**
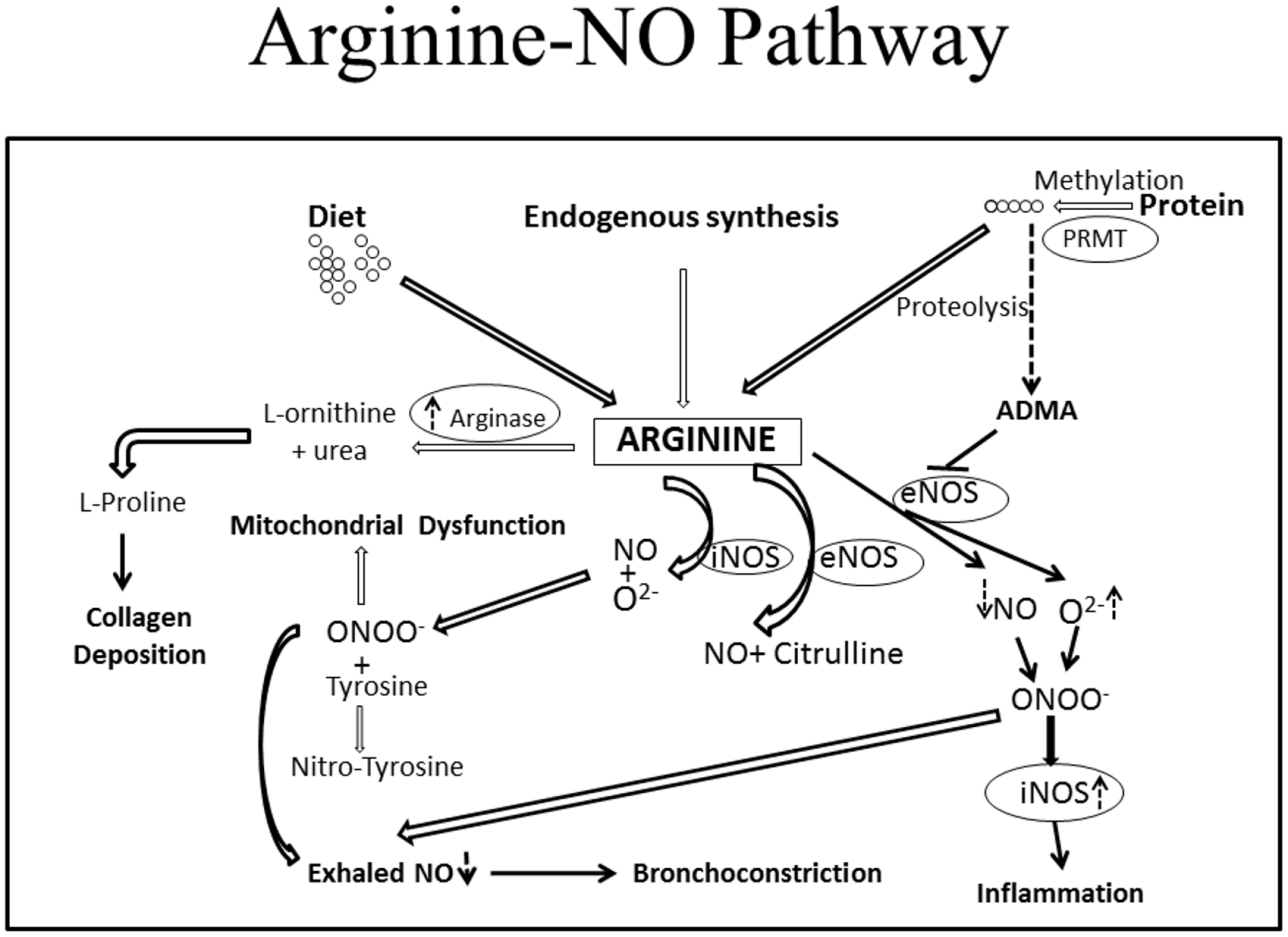
Arginine-NO pathway.

The principal finding is that the metabolic syndrome in non-obese HFR mice was associated with increased baseline lung resistance, airway hyperresponsiveness, and decreased exhaled NO. This was similar to observed changes in obese HFA mice except that during methacholine challenge, the increase in lung resistance was greater in obese (HFA) mice than those with only metabolic syndrome (HFR). Also, a large increase in elastance was seen exclusively in the HFA group. This is suggestive of derecruitment of peripheral lung units and it is likely that the constant 2 cm H_2_O PEEP during ventilation was sufficient to prevent peripheral airway closure in normal weight mice (HFR and control) but not obese mice (HFA). In support, while there was a possible increase in collagen content in lungs of both HFA and HFR mice, there was no significant parenchymal abnormality in either group. Irrespective of these differences between HFA and HFR mice, the increased resistance to airflow, with low levels of exhaled NO and lack of inflammatory cell infiltrate, is very similar to the “obese-asthma” phenotype in humans [30]. It is likely that this obese-asthmatic phenotype could be driven by abnormal Arginine-NO metabolism, as suggested by Holguin et al and us [12,33,31]. We have previously suggested that ADMA is a common link between asthma and metabolic syndrome [12]. While ADMA can bind to NOS in place of L-arginine, the methyl group prevents transfer of electrons to the nitrogen, preventing formation of NO and leading to formation of superoxide anion. NO generated from iNOS, which is relatively resistant to ADMA, can react with superoxide to form peroxynitrite, which can lead to protein nitrosylation and organelle damage. In obese HFA mice, there was additionally an increase in arginse expression and reduced levels of L-arginine, which by virtue of lowering arginine bioavailability could explain the greater AHR in such mice. Similar increase in ADMA and oxo-nitrative stress has previously been reported in allergic asthma [33,43,40] and we speculate that this could be a major mechanism, unrelated to any classical immune response, which may be responsible for the obese-asthma phenotype such that airway stress and hyperresponsiveness develop without any cellular inflammation. We have previously shown that such mice have evidence of mitochondrial derangement and Fig 7 shows a schematic of how the current findings may explain that observation [44, 45].

In summary, diet-induced metabolic changes similar to that seen in obesity has significant local effects on the lungs and induces asthma-like changes, even without increased body mass or adiposity. Increased ADMA and consequent oxo-nitrative stress are one of the likely mechanisms. Other factors such as hyperinsulinemia, insulin resistance [46], cytokines, amongst others, may be additionally important. Other mechanisms, such as susceptibility to peripheral airway closure through mechanical effects or effects of adipose hormones, may be additionally relevant in obese subjects. This should be further explored in longitudinal studies of human subjects with metabolic syndrome and normal or increased BMI.

## Supporting Information

S1 Fig Immunohistochemistry profiling shows increase in arginase I expression. Immunohistochemistry profiling of arginase I expression. The splitting of two images for DAB and Hematoxylin expression differentiation. Brown colour (DAB) indicates positive expression and Blue (Hematoxylin) colour shows stained nuclei. Representative images are shown from each group. All photographs are at 10X magnification.
(TIF)

S2 Fig Immunohistochemistry profiling shows increase in iNOS expression. Immunohistochemistry profiling of iNOS expression. The splitting of two images for DAB and Hematoxylin expression differentiation. Brown colour (DAB) indicates positive expression and Blue (Hematoxylin) colour shows stained nuclei. Representative images are shown from each group. All photographs are at 10X magnification.
(TIF)

S3 Fig Collagen content by Masson’s Trichrome Staining. (A) Sub epithelial collagen was stained by Masson Trichrome staining in lung tissue sections Representative images are shown from each group. All photographs are at 10X magnification. Scale bar = 100μm. (B) Collagen content was estimated by quantitative morphometry.
(TIF)
