# Supplementary figures and images for: Metabolic Syndrome Is Associated with Increased Oxo-Nitrative Stress and Asthma-Like Changes in Lungs
Source: PLoS One. 2015 Jun 22;10(6):e0129850. doi: 10.1371/journal.pone.0129850 (PMC4476757; doi:10.1371/journal.pone.0129850)

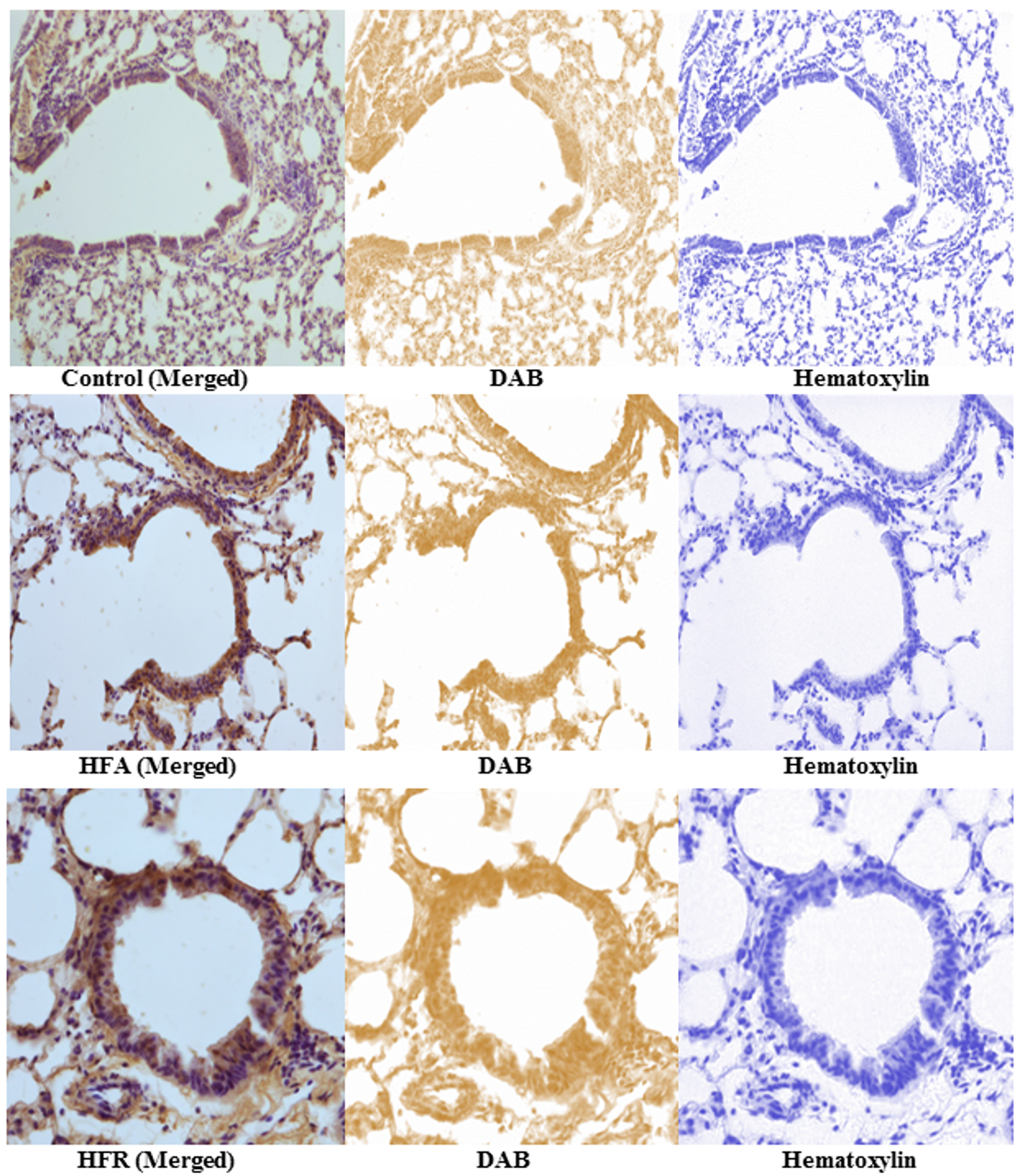

Supplement: S1 Fig — Immunohistochemistry profiling of arginase I expression. The splitting of two images for DAB and Hematoxylin expression differentiation. Brown colour (DAB) indicates positive expression and Blue (Hematoxylin) colour shows stained nuclei. Representative images are shown from each group. All photographs are at 10X magnification. (TIF) [file pone.0129850.s001.tif]

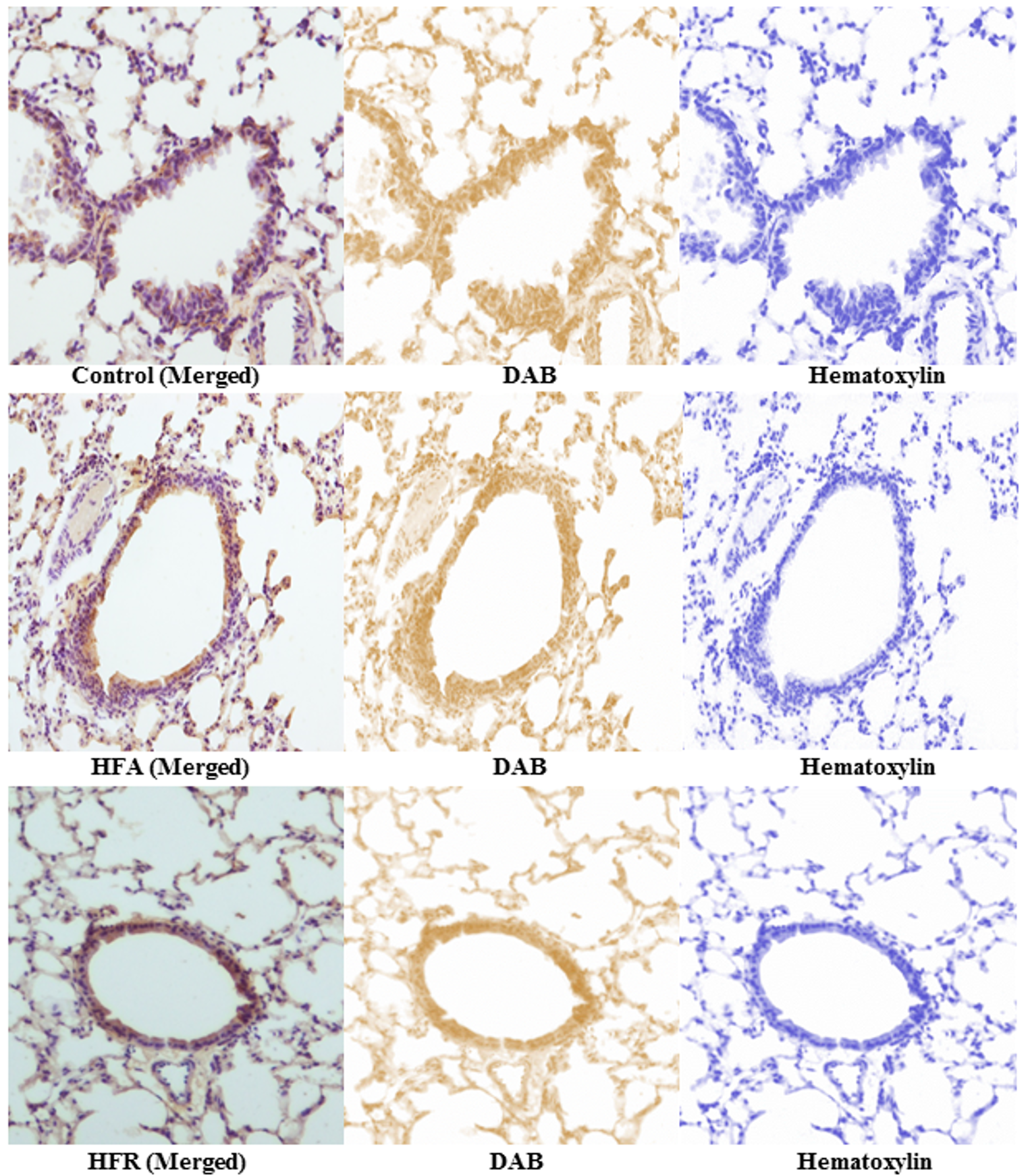

Supplement: S2 Fig — Immunohistochemistry profiling of iNOS expression. The splitting of two images for DAB and Hematoxylin expression differentiation. Brown colour (DAB) indicates positive expression and Blue (Hematoxylin) colour shows stained nuclei. Representative images are shown from each group. All photographs are at 10X magnification. (TIF) [file pone.0129850.s002.tif]

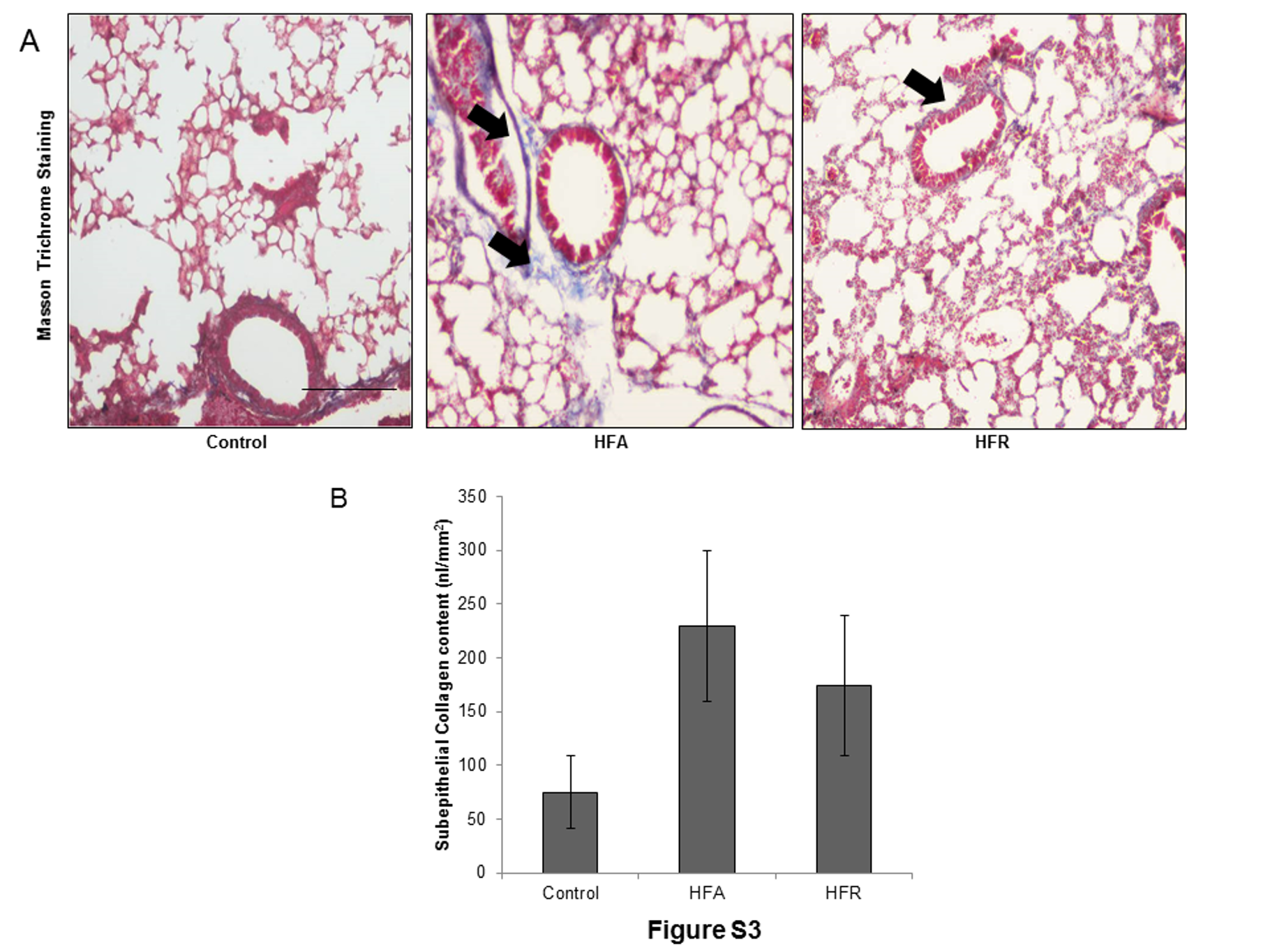

Supplement: S3 Fig — (A) Sub epithelial collagen was stained by Masson Trichrome staining in lung tissue sections Representative images are shown from each group. All photographs are at 10X magnification. Scale bar = 100μm. (B) Collagen content was estimated by quantitative morphometry. (TIF) [file pone.0129850.s003.tif]
